# Self-Compassion May Have Benefits for Body Image among Women with a Higher Body Mass Index and Internalized Weight Bias

**DOI:** 10.3390/healthcare11070970

**Published:** 2023-03-29

**Authors:** Bethany A. Nightingale, Stephanie E. Cassin

**Affiliations:** 1 Department of Psychology, Toronto Metropolitan University, 350 Victoria St, Toronto, ON M5B 2K3, Canada; 2 Department of Psychiatry, University of Toronto, 250 College St. (8th floor), Toronto, ON M5T 1R8, Canada; 3 Centre for Mental Health, University Health Network, 190 Elizabeth St., Toronto, ON M5G 2C4, Canada

**Keywords:** body image, body weight, compassion, self-compassion, weight bias internalization

## Abstract

Negative attitudes towards one’s own body are common among women and are linked to adverse consequences including negative affect, low self-esteem, and eating pathology. Self-compassion has been found effective in improving body image; however, few published studies have examined self-compassion in populations with higher BMIs despite the positive correlation between weight and body dissatisfaction. The current study examined the efficacy of a self-compassion letter-writing exercise versus two active control groups in response to a negative body image induction. The sample of college-aged females (*M* age = 20.91 years; *SD* = 5.47) was split between higher and lower BMI to determine whether self-compassion affects body image, affect, and self-esteem differently across weight groups. Weight bias internalization (WBI: i.e., internalization of society’s negative stigma against those with higher BMIs) was examined as a moderator of this relationship in the higher BMI group. Results suggest that letter writing improved body image regardless of condition (*p* < 0.001). The self-compassion exercise promoted more adaptive body image (*p* = 0.007) and self-compassion (*p* = 0.013) than one control condition for those with high WBI. Results suggest that self-compassion can be helpful in ameliorating negative body image for females of all sizes, and that levels of WBI may alter the effect of body image interventions.

## 1. Introduction

Negative body image is so common among women in Western culture that it has been deemed “normative discontent” [1]. In general, modern Western culture promotes a thin figure as the ideal for women, and weight can play a large role in women’s thoughts and feelings towards their own bodies [2]. Media inundates consumers with images of the “perfect” woman and, as Rodin and colleagues [1] (p. 269) wrote, “legions of women pursue thinness like a career.” The relationship between media consumption and body image has received a great deal of empirical support across the decades. Bessenoff [3], Fallon, and Hausenblas [4], and Harper and Tiggemann [5], for example, found that women observing media depiction of the “thin ideal” had greater body dissatisfaction than women observing a control advertisement. Thompson and Stice [6], as well as Stice and Shaw [7], propose that internalizing this ideal is related to greater body dissatisfaction. Recent studies have found that viewing messages promoting *any* type of body ideal (e.g., thin, athletic, curvy) prompts self-objectification [8], that is, the internalization and application of societal objectification of women’s bodies to oneself [9]. This finding speaks to the possibility that the relationship between body ideals and body image-related constructs may be stable despite the changing nature of the ideals themselves (e.g., thin versus curvy).

Negative body image has a plethora of negative consequences. Experiencing body dissatisfaction renders young women more susceptible to eating disorder symptomatology (e.g., [10]), and has also been linked to various other harmful outcomes such as depressed mood [11], low self-esteem [11], certain risky behaviors such as using drugs [12], engagement in self-harm [12], and even attempted suicide [13]. Given the prevalence and adverse effects of negative body image, it is important to develop and examine the impact of interventions aimed at improving body image. One genre of body image interventions that has received recent attention is self-compassion (e.g., [14,15,16,17]). Self-compassion is an attitude taken towards oneself in the face of suffering or distress [18]; in short, it is simply compassion turned inward towards oneself. Self-compassion, as defined by Neff [18], is composed of three separate parts. The first component is *self-kindness*, or being warm and understanding towards oneself in the face of distress or failure, in contrast to *self-judgement*, which entails being harsh and critical towards oneself for any shortcoming. The second component is *common humanity*, which involves recognizing that suffering and failure are part of what it means to be human, versus *isolation*, which involves thoughts that one is the only person with a certain negative experience or emotion. The third component involves *mindfulness*, or recognizing one’s thoughts and feelings without *over-identifying* with them or avoiding them. Self-compassion has been linked to many positive self-attitudes and outcomes, such as higher self-esteem (e.g., [19]) and more adaptive affect (e.g., [20]).

Self-compassion has been found to be negatively associated with body dissatisfaction [21] and body shame [22], and positively associated with body appreciation [23], making it a good candidate for a body image intervention strategy. In experimental designs, self-compassion has been found to improve body satisfaction. For example, Slater and colleagues [24] found that, compared to women viewing decoration photos, women with average-to-high trait thin-ideal internalization who were exposed to Instagram photos with self-compassion-themed quotes had increased body satisfaction over the study period. Similarly, women who engaged in daily self-compassion meditations for three weeks had larger decreases in body dissatisfaction than women in a wait-list control group [14], and engaging in at least one self-compassion training exercise has been linked to improvements in several body image-related variables after one week [15].

Previous research investigating the impact of self-compassion interventions on body image has largely been conducted with samples of women with more average weight and/or combined samples of persons with various BMIs; however, those with higher BMIs have higher body dissatisfaction on average than those with weight in the normal BMI range (i.e., 18.5 kg/m^2^ to 24.9 kg/m^2^; [25]). The limited research conducted to date among those with higher BMIs suggests that self-compassion may be helpful in improving body satisfaction. For example, David [16] found self-compassion to be as effective as cognitive restructuring in improving body image among women with higher BMIs. Forbes and colleagues [17] found that two days of intensive compassion-focused therapy was helpful in ameliorating body dissatisfaction among women with higher BMIs. Haley and colleagues [26] found in their recent pilot study that a three-week-long intervention was helpful in ameliorating body appreciation, but not body image shame, among women with higher BMIs and weight bias internalization.

It is important to consider that weight bias internalization may influence the effectiveness of self-compassion interventions. Weight bias, in general, refers to stigma against those with higher BMIs (e.g., [27]). These individuals are perceived as being unhealthy, lazy, unattractive, un-athletic, and unhappy, and as having poor hygiene [28]. Individuals with higher BMIs may internalize these societal beliefs as being true of themselves [29], a process referred to as weight bias internalization (WBI).

Higher WBI is associated with many negative outcomes, including negative body image [30,31], poor self-esteem (e.g., [30,31]), anxiety [32], depression (e.g., [30,33]), stress [30], and negative affect [31]. In addition, WBI is negatively correlated with self-compassion (e.g., [34]). One study conducted by Hilbert and colleagues examined the role of self-compassion in the relationship between WBI and outcomes such as health-related quality of life, depression, and somatic symptoms in individuals with higher BMIs [35]. Self-compassion mediated the relationship between these variables, such that when trait self-compassion was present, the direct positive relationships between WBI and depression/somatic symptoms, as well as the negative relationship between WBI and health-related quality of life, were attenuated [35]. This finding indicates that self-compassion may be an effective strategy in attenuating certain types of distress in populations with higher BMIs.

Previous research has not examined whether the effect of state (or induced) self-compassion varies depending on an individual’s level of WBI. Since those with high WBI seem to have lower self-compassion [34], self-compassion interventions may not have the same benefits in those who have internalized society’s bias against those with higher BMIs. Self-compassion may be “blocked”, so to speak. Conversely, as suggested by Hilbert and colleagues’ findings [35], learning about and practicing self-compassion in the face of negative body image may be particularly freeing for those with high WBI. To our knowledge, this question has not yet been examined empirically, and thus it remains unknown whether self-compassion is a helpful strategy among individuals with higher BMIs and high WBI.

The current study investigated the effect of a self-compassion exercise on body satisfaction/dissatisfaction over several time points, as well as the effect on body image, affect, self-compassion, and self-esteem in comparison to two control groups (positive thinking or neutral control exercises), and whether these effects differed in individuals with higher versus lower BMIs. On a more exploratory basis, it also examined whether WBI moderated the relationship between self-compassion and the outcome variables for those with higher BMIs (i.e., whether practicing self-compassion had a different effect on those with high versus low weight bias internalization). The hypotheses were as follows:

(1) Those completing the self-compassion exercise will report significant increases in body satisfaction and decreases in body dissatisfaction over time, whereas those completing the positive thinking and neutral control exercises will have non-significant differences between these time points, in both those with higher and lower BMIs.

(2) Those completing the self-compassion exercise will report higher self-compassion, self-esteem, positive body image, and positive affect as well as lower negative affect than those completing the positive thinking and neutral control exercises, in both those with higher and lower BMIs.

## 2. Materials and Methods

### 2.1. Participants

Undergraduate students (N = 168) were recruited through a subject pool at a Canadian university. For a power of 0.8 using medium effect sizes and an alpha value of 0.01, 227 individuals were needed (G*Power [36]). However, due to extenuating circumstances (the beginning of the COVID-19 pandemic in March 2020), recruitment halted and only 168 participants completed the study. However, lower power can provide more confidence that any significant effects actually exist. Participation was limited to females because body dissatisfaction appears to present differently in males [37,38]. Stratified sampling was used whereby participants were recruited based on their self-reported weight in a pre-screen measure. This sampling strategy resulted in approximately half of the participants having a BMI ≥ 25 kg/m^2^ and the other half having a BMI ≤ 25 kg/m^2^. Participants had a mean age of 20.91 years (*SD* = 5.47, range = 17–52) and a mean BMI of 25.60 kg/m^2^ (*SD* = 5.08, range = 15.10–50.30). The sample was quite diverse. Participant characteristics as a function of weight group (“lower BMI”, “higher BMI”) and letter-writing condition and are presented in Table 1.

### 2.2. Measures

#### 2.2.1. Body Mass Index (BMI)

BMI, a standardized measure of body weight, is calculated based on an individual’s weight and height and is typically divided into four categories: underweight, normal weight, overweight, and obese (e.g., [39]). For the purposes of this study, we used the cut-off of 25 kg/m^2^ suggested by the National Heart, Lung, and Blood Institute [39] to be the threshold between normal and overweight, and divided the sample into a “higher BMI” group and a “lower BMI” group. However, there are several issues with the use of BMI as a categorization tool, as it does not include the various factors that influence weight or outcomes of weight, such as muscle mass, bone mass, ethnic background [40], age, fat mass, distribution of body fat throughout the body, and changes in population height and weight over time [41]. We chose to use BMI, including its typical ‘cut-off’ categories, as an indicator of weight status as it remains a tool for rough estimation of body size which is used by many health professionals; however, results should be interpreted in light of these measurement limitations.

#### 2.2.2. Modified Weight Bias Internalization Scale (WBIS-M [42])

The WBIS-M is an 11-item self-report questionnaire that assesses the internalization of weight bias in individuals of all BMIs and is a modification of the original scale designed for persons with a BMI over 25 kg/m^2^ [43]. Participants were asked to indicate their level of agreement with each item on a seven-point Likert scale, with anchors labeled “strongly disagree” and “strongly agree” (e.g., “Because of my weight, I feel that I’m just as competent as anyone”; “I am less attractive than most people because of my weight”). The internal consistency of the WBIS was originally found to be 0.94 [42] and was 0.93 in the present sample. It has also demonstrated good convergent validity; it is related to the Body Shape Questionnaire, binge eating in men and women, and the drive for thinness in women, among other questionnaires [42].

#### 2.2.3. Visual Analogue Scales (VAS) for Body Satisfaction and Dissatisfaction

Body satisfaction and dissatisfaction were measured at various time points. The two VAS items ask participants to rate how they feel about their body on two hundred-point scales with anchors ranging from No Satisfaction/Dissatisfaction to Extreme Satisfaction/Dissatisfaction. VAS have been shown to be effective in measuring a variety of constructs (e.g., anxiety [44]) including body satisfaction/dissatisfaction [45]. In fact, VAS measuring weight and appearance dissatisfaction correlated at 0.66 and 0.76 with the body dissatisfaction subscale of the Eating Disorders Inventory [45,46]. The particular VAS used in the present study have also been used in previous experimental research examining the impact of self-compassion on body image among women [16].

#### 2.2.4. International Positive and Negative Affect Schedule-Short Form (I-PANAS-SF [47])

The I-PANAS-SF is a 10-item short form of a scale created to assess positive affect (PA) and negative affect (NA) on a five-point scale [48]. This scale is moderately correlated with subjective well-being and happiness [47]. The I-PANAS-SF instructions were reworded for the current study to reflect state affect (“Thinking about yourself and how you feel right now” as opposed to “how you normally feel”). Internal consistency in the current sample was good, with a Cronbach’s alpha of 0.85 for NA and 0.77 for PA.

#### 2.2.5. Self-Compassion Scale (SCS [18])

The SCS is a 26-item self-report measure of self-compassion. Respondents are asked to rate each item on a five-point scale (e.g., “When something painful happens I try to take a balanced view of the situation”). For the purposes of the present study, respondents were asked to respond to the items in reference to their feelings about a negative body image scenario (i.e., state self-compassion rather than trait self-compassion), and anchors were changed accordingly (i.e., Not at All/Extremely instead of Almost Never/Almost Always). Higher scores on this scale indicate greater self-compassion. Full scale scores were used in all analyses. Neff [18] found the scale to be correlated with social connectedness and self-criticism [18], providing evidence for convergent validity. The SCS has excellent internal consistency, with a Cronbach’s alpha of 0.89 in the present sample.

#### 2.2.6. Body Image States Scale (BISS [49])

The BISS is a 6-item self-report measure that assesses state body image. Respondents are asked to rate their current feelings towards their body on a nine-point Likert scale on anchors such as Extremely Dissatisfied/Extremely Satisfied, which vary according to the particular item (e.g., “Right now I feel *extremely dissatisfied* with my physical appearance”; “Right now I feel *a great deal worse* about my looks than I usually feel” [49]). Higher scores on this scale indicate more positive body image. Internal consistency was originally found to range between 0.72 and 0.77 [49] and was 0.83 in the present study. The BISS has also demonstrated good validity; for example, those with higher “dysfunctional investment in their appearance” [49] (p. 108) had more negative body image than those who were lower on this trait [49].

#### 2.2.7. State Self-Esteem Scale (SSES [50])

The SSES is a 20-item self-report measure that assesses state fluctuations in self-esteem. Respondents are asked to rate each item on a five-point scale ranging from Not at All to Extremely (e.g., “I feel confident that I understand things”; “I feel others respect and admire me”). Higher scores on this scale indicate greater self-esteem. The scale demonstrated excellent internal consistency, with an alpha of 0.92 in the original psychometric study [50] and 0.93 in the current study. It has also demonstrated good validity as it is positively correlated with trait self-esteem and negatively correlated with depression and anxiety [50].

### 2.3. Procedures

Upon arrival at the lab, participants provided consent and completed Visual Analogue Scales (VAS) measuring their body satisfaction/dissatisfaction, a demographic questionnaire, and the WBIS-M online via Qualtrics. Participants were subsequently weighed and asked to complete the same VAS again. Subsequently, participants wrote about a recent situation during which they felt poorly about their body (e.g., noticing a body part they dislike while looking in the mirror), a step intended to make negative body image salient. The instructions were partially based on negative mood inductions found to be effective in previous research [20,51]. After writing, participants completed VAS body (dis)satisfaction scales for a third time. Participants were then randomized to a self-compassion (experimental group), positive thinking (active control group), or neutral control condition. They were asked to write a letter that was either a self-compassionate or optimistic reaction to their negative body image scenario, or to write about their day’s activities (similar to control conditions used by David [16] and Gonen [52]).

The self-compassion letter-writing exercise was informed by Neff’s conceptualization of self-compassion [18], and by self-compassionate writing exercises used by Przezdziecki [20], David [16], Ziemer and colleagues [53], and Kelly and Waring [54]. Participants were instructed to write a compassionate letter to themselves communicating themes of self-kindness, common humanity, and mindfulness. The positive thinking letter-writing exercise asked participants to write an upbeat letter to themselves about the situation, communicating themes of optimism, “looking on the bright side”, and remembering that they have other things to be happy about. Positive thinking was selected as an active control because it is a seemingly credible and helpful way of thinking about body-related distressing events. The neutral control letter-writing exercise simply asked participants to write a letter to themselves describing their schedule for the remainder of the day. The letter-writing exercise instructions are available from the corresponding author upon request. After finishing the letter, participants completed dependent variable measures (VAS, SCS, BISS, SSES, and I-PANAS-SF), read a debriefing form, and watched a DOVE self-esteem video intended to boost body satisfaction. All procedures were approved by the institution’s Research Ethics Board: REB # 2019-311.

## 3. Results

### 3.1. The Impact of the Letter-Writing Exercises on Body (Dis)Satisfaction

Two 4 × 3 × 2 mixed ANOVAs were conducted with body (dis)satisfaction VAS scores as the dependent variables to determine whether body dis(satisfaction) changed over time (T1: baseline; T2: following weighing; T3: following negative body image induction; T4: following letter-writing exercise) as a function of letter-writing condition (self-compassion; positive thinking; neutral control), and weight group (lower BMI; higher BMI).

Omnibus results using the Greenhouse–Geisser correction demonstrated that there were no three-way interactions between weight group, letter-writing condition, and time (body satisfaction, *F*(4.93,372.13) = 1.58, *p* = 0.17, ηp^2^ = 0.02; body dissatisfaction, *F*(4.45,342.24) = 1.46, *p* = 0.21, ηp^2^ = 0.02), or two-way interactions between time and weight group (body satisfaction, *F*(2.46, 372.13) = 2.11, *p* = 0.11, ηp^2^ = 0.01; body dissatisfaction, *F*(2.22, 342.24) = 1.55, *p* = 0.21, ηp^2^ = 0.01) or time and letter-writing condition (body satisfaction, *F*(4.93, 372.13) = 0.88, *p* = 0.49, ηp^2^ = 0.01; body dissatisfaction, *F*(4.45, 342.24) = 1.47, *p* = 0.21, ηp^2^ = 0.02). Omnibus between-subject results indicated no two-way interactions between weight group and letter-writing condition (body satisfaction, *F*(2,151) = 0.24, *p* = 0.79, ηp^2^ = 0.003; body dissatisfaction, *F*(2,154) = 1.62, *p* = 0.20, ηp^2^ = 0.02).

Similarly, omnibus main-effect results indicated no effect of letter-writing condition on either dependent variable (body satisfaction, *F*(2,151) = 1.45, *p* = 0.24 ηp^2^ = 0.02; body dissatisfaction, *F*(2,154) = 2.31, *p* = 0.103, ηp^2^ = 0.03). However, the omnibus main effect of weight group was significant for body satisfaction, *F*(1,151) = 13.31 *p* < 0.001, ηp^2^ = 0.08, and dissatisfaction, *F*(1,154) = 8.37, *p* = 0.004, ηp^2^ = 0.05. The omnibus within-subject main effects for time suggested body satisfaction, *F*(2.46, 372.13) = 21.56, *p* < 0.001, ηp^2^ = 0.13, and dissatisfaction, *F*(2.22, 342.24) = 24.93, *p* < 0.001, ηp^2^ = 0.14, significantly changed over time at a Bonferroni-corrected alpha level of 0.03. Follow-up one-way ANOVAs using a Bonferroni-corrected alpha value of 0.01 for the main effects of weight group indicated that body satisfaction was significantly higher among those in the lower BMI group (*overall M* = 62.89) than among those in the higher BMI group (*overall M* = 49.96) at all time points (T1, *F*(1,160) = 13.93, *p* < 0.001; T2, *F*(1,159) = 16.62, *p* < 0.001; T3, *F*(1,159) = 11.59, *p* = 0.001; T4, *F*(1,162) = 7.77, *p* = 0.006). Similarly, body dissatisfaction was significantly lower among those in the lower BMI group (*overall M* = 41.73) than among those in the higher BMI group (*overall M* = 53.16) at T2, *F*(1,163) = 10.22, *p* = 0.002, T3, *F*(1,164) = 9.03, *p* = 0.003, and T4, *F*(1,163) = 9.81, *p* = 0.002, but not at T1, *F*(1,162) = 3.69, *p* = 0.057.

Follow-up paired-sample t-tests using a Bonferroni-corrected alpha value of 0.008 for the main effects of time indicated that body satisfaction was significantly lower at T2 (*M* = 53.18; *SD* = 24.41) than at T1 (*M* = 57.75; *SD* = 22.62), *t*(158) = 4.10, *p* < 0.001), and body dissatisfaction was significantly higher at T2 (*M* = 51.50; *SD* = 26.97) than at T1 (*M* = 46.40; *SD* = 24.76), *t*(163) = −4.01, *p* < 0.001). Although T3 body satisfaction (*M* = 53.44; *SD* = 24.91) was also significantly lower than baseline, *t*(157) *=* 3.80, *p* < 0.001, and T3 body dissatisfaction (*M* = 52.16, *SD* = 27.24) was higher than baseline, *t*(163) = −4.55, *p* < 0.001, the differences in body (dis)satisfaction between T2 and T3 were non-significant, *p*s > 0.426. These results indicate that weighing participants worsened their body (dis)satisfaction and that this effect was maintained after the negative body image induction.

**Figure 1:**
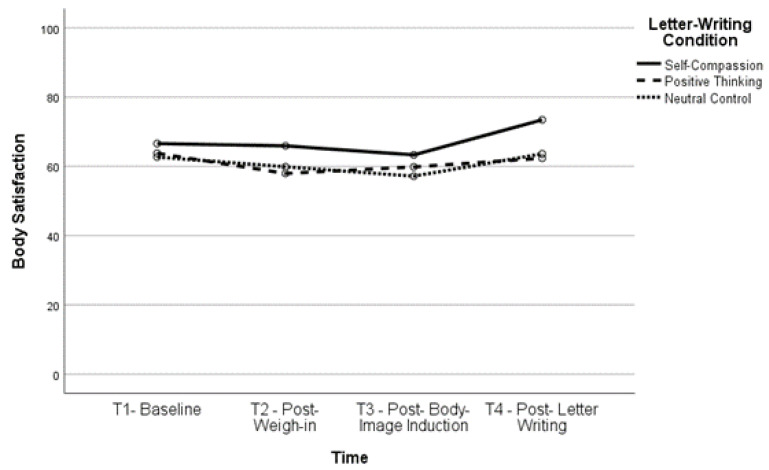
Body Satisfaction Over Time for Lower BMI Group.

**Figure 2:**
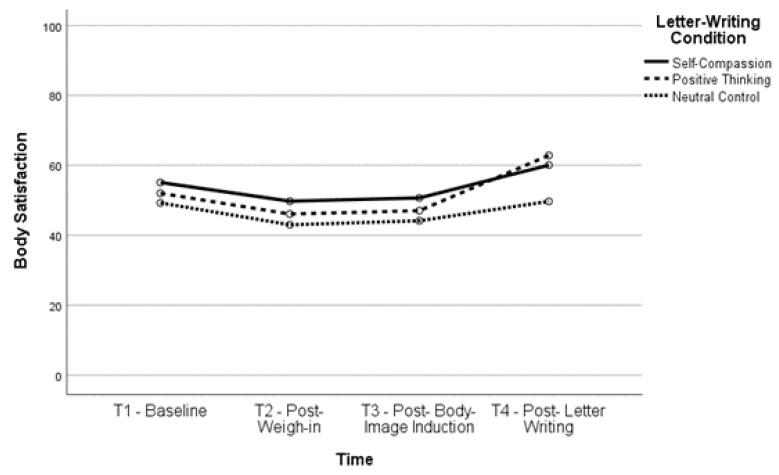
Body Satisfaction Over Time for Higher BMI Group.

**Figure 3:**
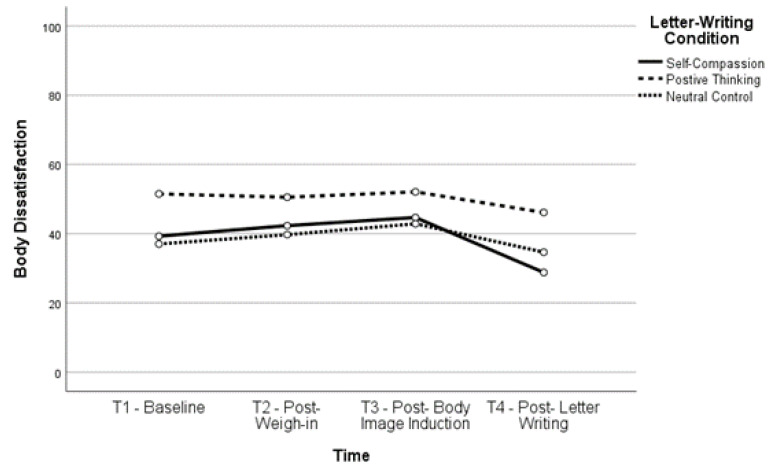
Body Dissatisfaction Over Time for Lower BMI Group.

**Figure 4:**
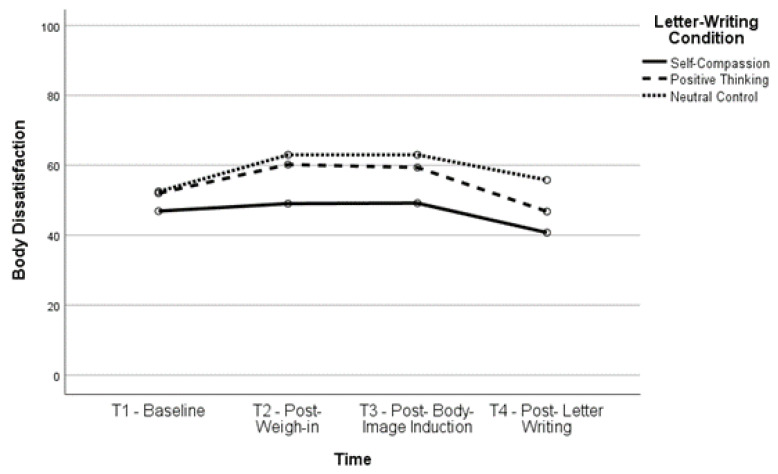
Body Dissatisfaction Over Time for Higher BMI Group.

Body satisfaction significantly increased, *t*(160) = −6.12, *p* < 0.001, and body dissatisfaction significantly decreased, *t*(163) = 6.01, *p* < 0.001, between T2 and T4 (body satisfaction, *M* = 61.52, *SD* = 23.54; body dissatisfaction, *M* = 42.39, *SD* = 26.22) as well as between T3 and T4 (body satisfaction, *t*(161) = −6.35, *p* < 0.001; body dissatisfaction, *t*(164) = 6.51, *p* < 0.001), indicating that body (dis)satisfaction improved after completing the letter-writing exercises. In fact, both body satisfaction, *t*(160) = −2.93, *p* = 0.004, and body dissatisfaction, *t*(162) = 3.03, *p* = 0.003, were significantly improved at T4 compared to baseline, indicating that the letter-writing exercises not only ameliorated the impact of the negative body image induction but also increased positive body image beyond what participants reported at baseline. See Figure 1, Figure 2, Figure 3 and Figure 4 for changes in body satisfaction and dissatisfaction across the experimental procedure.

### 3.2. Group Differences following the Letter-Writing Exercise

To test the hypothesis that the self-compassion group would have more adaptive scores on the dependent variables regardless of participant weight, a 2 (participant weight) × 3 (experimental condition) ANOVA was conducted separately for each of the five remaining dependent variables (self-compassion, self-esteem, positive affect, negative affect, body image).

Mean scores for each of the dependent variables are presented in Table 2. Contrary to hypotheses, the main effect of letter-writing condition was not significant for any of the dependent variables at a Bonferroni-corrected level of 0.01 (self-compassion, *F*(2,158) = 2.69, *p* = 0.071, ηp^2^ = 0.03; self-esteem, *F*(2,161) = 1.008, *p* = 0.371, ηp^2^ = 0.01; body image, *F*(2,160) = 2.09, *p* = 0.127, ηp^2^ = 0.03; positive affect, *F*(2,158) = 1.55, *p* = 0.215, ηp^2^ = 0.02; negative affect, *F*(2,157) = 0.28, *p* = 0.759, ηp^2^ = 0.00). Similarly, the interaction between letter-writing condition and participant weight was not significant for any of the dependent variables (self-compassion, *F*(2,158) = 0.83, *p* = 0.440, ηp^2^ = 0.01; self-esteem, *F*(2,161) = 0.11, *p* = 0.89, ηp^2^ = 0.00; body image, *F*(2,160) = 0.48, *p* = 0.615, ηp^2^ = 0.01; positive affect, *F*(2,158) = 0.39, *p* = 0.679; ηp^2^ = 0.01; negative affect, *F*(2,157) = 1.04, *p* = 0.358, ηp^2^ = 0.01). These findings suggest that brief practice in self-compassion did not have significant benefits in comparison to the control conditions, regardless of participant weight status.

### 3.3. Exploratory Analysis

Hierarchical regressions were conducted to examine whether WBI moderated the relationship between letter-writing condition and the dependent variables for those with higher BMIs. For each regression, WBI and dummy-coded variables contrasting letter-writing conditions were entered as the first level of the hierarchical regression. For five of the regressions, the dummy codes contrasted the self-compassion and positive thinking letter-writing conditions against the neutral control letter-writing condition for all dependent variables; the remaining five regressions had dummy codes contrasting the self-compassion and neutral control letter-writing conditions against the positive thinking letter-writing condition. Two interaction terms (WBISM*dummy coded variables) were calculated and added as the second level of the analysis for each regression.

Significant results were followed up by re-running the regressions with WBI scores centered one standard deviation above the mean, on the mean, and one standard deviation below the mean, respectively. These analyses provided simple slopes for the effect of letter-writing condition on the dependent variables at high, medium, and low levels of WBI. To this end, a total of six regressions were conducted for each dependent variable. The negative affect data were heteroscedastic, so a weighted least squares regression was conducted to analyze these data, as suggested by Field [55].

Hierarchical regressions exploring whether WBI changes the relationship between letter-writing condition and dependent variables among those with higher BMIs were conducted despite the lack of significant association between condition and dependent variables, because it was possible for the relationship to *become* significant at certain levels of weight bias internalization. Therefore, exploratory analyses were conducted as planned.

**Figure 5:**
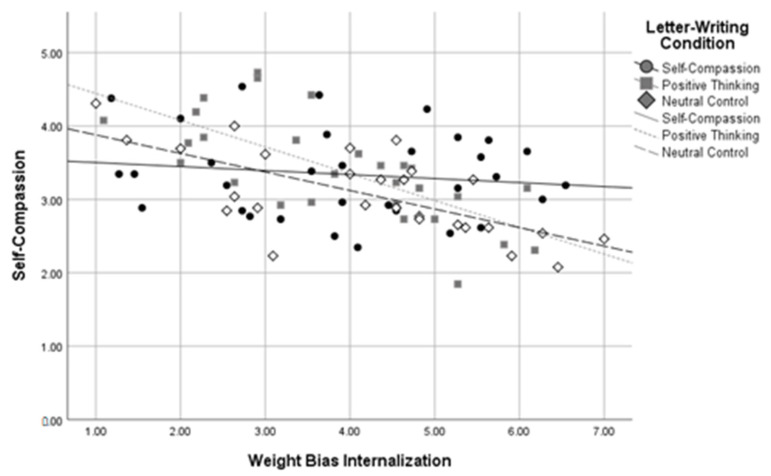
Letter-Writing Condition*Weight Bias Internalization Interaction on Self-Compassion.

**Figure 6:**
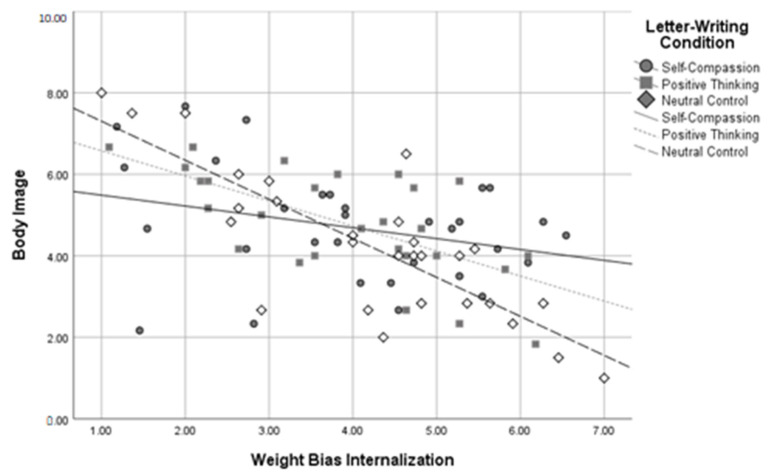
Letter-Writing Condition*Weight Bias Internalization Interaction on Body Image.

**Figure 7:**
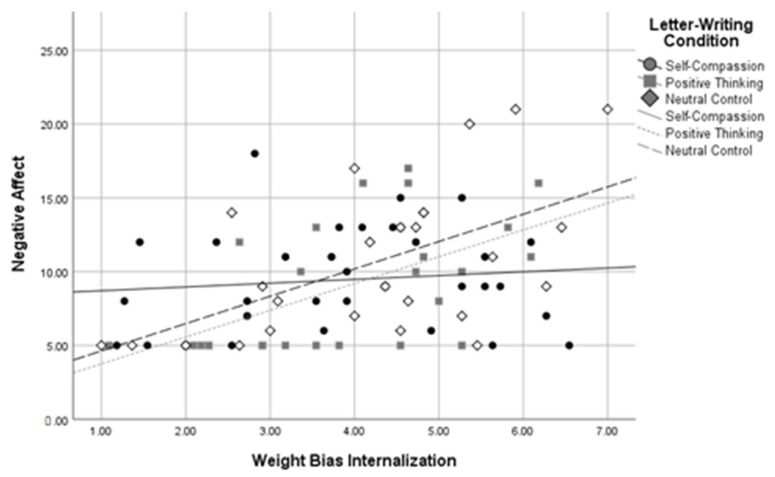
Letter-Writing Condition*Weight Bias Internalization Interaction on Negative Affect.

Using dummy codes contrasting the self-compassion and positive thinking conditions against the neutral control condition, *R*^2^ *change* between the two levels of the model was significant when predicting self-compassion, *R*^2^ *change* = 0.09, *F change*(2,81) = 5.62, *p* = 0.005, body image, *R*^2^ *change* = 0.08, *F change*(2,81) = 6.13, *p* = 0.003, and negative affect, *R*^2^ *change* = 0.08, *F change*(2,79) = 5.06, *p* = 0.009, at a Bonferroni-corrected alpha of 0.01. The *R*^2^ *change* between the two levels of the model was not significant when predicting self-esteem, *R*^2^ *change* = 0.03, *F change*(2,82) = 2.60, *p* = 0.080, or positive affect, *R*^2^ *change* = 0.04, *F change*(2,80) = 1.86, *p* = 0.162. Using dummy codes contrasting the self-compassion and neutral control conditions against the positive thinking condition, *R*^2^ *change* between these two levels of the model was again significant when predicting self-compassion, *R*^2^ *change* = 0.09, *F change*(2,81) = 5.48, *p* = 0.006, body image, *R*^2^ *change* = 0.08, *F change*(2,81) = 6.18, *p* = 0.003, and negative affect, *R*^2^ *change* = 0.08, *F change*(2,79) = 5.05, *p* = 0.009, at a Bonferroni-corrected alpha of 0.01. The *R*^2^ *change* between the two levels of the model was not significant when predicting self-esteem, *R*^2^ *change* = 0.03, *F change* (2,82) = 2.60, *p* = 0.080, or positive affect, *R*^2^ *change* = 0.04, *F change*(2,80) = 1.80, *p* = 0.172. These results indicate that WBI and letter-writing condition interacted to predict self-compassion, body image, and negative affect in participants with higher BMIs. See Figure 5, Figure 6 and Figure 7 for interaction plots.

Three follow-up regressions were conducted for each of these significant interactions to determine the relationship between letter-writing condition and body image at high, medium, and low levels of WBI. Analyses used a Bonferroni-corrected alpha level of 0.017. The results indicated that those in the self-compassion condition had more positive body image than those in the neutral control condition, *β* = 0.98, *SE* = 0.361, *p* = 0.007, but only at high levels of WBI. A similar pattern emerged for levels of self-compassion, with those in the self-compassion condition reporting higher self-compassion than those in the neutral control condition, *β* = 0.41, *SE* = 0.16, *p* = 0.013, but only at high levels of weight bias internalization. In contrast, at low levels of WBI, those in the self-compassion condition reported *lower* self-compassion than those in the positive thinking condition, *β* = −0.59, *SE* = 0.22, *p* = 0.008. Similarly, those in the self-compassion condition reported higher negative affect scores than those in the positive thinking condition, *β* = 3.35, *SE* = 1.31, *p* = 0.013, but only at low levels of WBI. No other letter-writing condition regression coefficients were significant. These follow-up results suggest that self-compassionate letter writing was related to more positive body image and higher self-compassion than the neutral control, but only for those with high baseline WBI, and that positive thinking letter writing was related to greater self-compassion and less negative affect than self-compassionate letter writing, but only for those with lower baseline WBI.

## 4. Discussion

The current study examined whether a self-compassion exercise results in significant improvement in body (dis)satisfaction and significant benefits to body image and related constructs, as well as whether levels of WBI have an impact on outcome variables for those with higher BMIs. There was mixed support for the study hypotheses. Contrary to the first hypothesis, self-compassion letter writing did not have significant benefits on most outcome variables; however, body (dis)satisfaction as measured by VAS scales indicated that body image improved after responding to the negative body image induction with letter writing, regardless of condition. The exploratory analyses indicated that those who wrote a self-compassionate letter reported more positive body image and self-compassion than those in the neutral control condition, but only amongst those who had higher baseline WBI. Additionally, those who wrote a positive thinking letter had higher self-compassion and lower negative affect than those who wrote a self-compassionate letter, but only amongst those who had lower levels of WBI.

The null effect of letter-writing condition was surprising given previous research indicating that self-compassion interventions can have an ameliorating effect on affect (e.g., [56]), self-compassion (e.g., [14]), and body image [57] compared to control conditions. Many previous studies examining the effect of self-compassionate letters on affect have had participant samples that were predominantly White (e.g., [53]) or left this demographic variable unreported (e.g., [20,56]) whereas the current study recruited and reported an ethnically diverse sample. It is possible that self-compassion interventions do not have the same effect for persons of color, however, some researchers (e.g., [24,58]) have reported beneficial effects of self-compassion with diverse samples. Some research demonstrates that self-compassion is differently related to self-criticism depending on ethnic group [59], while other studies find no differences in self-compassion between individuals of different ethnicities (e.g., [60]). These mixed findings suggest that ethnic differences in self-compassion are a possibility and may account in part for differences in the effect of self-compassion between samples.

The lack of significant benefit of the self-compassion letter-writing exercise in the present study may also be due to the brevity of the exercise. Some studies that demonstrated benefits used longer term exercises to induce self-compassion, such as daily self-compassion meditations for several weeks [14]. It is possible that the exercise used in the present study was too brief to have an immediate impact on outcome variables. The exercise format may also have impacted results; several studies finding significant effects of self-compassion used non-writing-based interventions such as social media/image-based or meditation-based interventions (e.g., [15]). It may be that writing-based exercises are not as helpful in fostering a self-compassionate mindset as are image- or meditation-based interventions. However, other studies examining brief self-compassion writing-based exercises have found them to be effective in improving affect, self-compassion, and/or body image (e.g., [20]) even after a negative body image induction [57,61], suggesting that the null results in the present study cannot be entirely attributed to the length or type of self-compassion exercise.

Importantly, the lack of significant benefit of the self-compassion letter-writing exercise could suggest that all three letter types were equally helpful in promoting self-compassion, self-esteem, positive body image, and positive affect. In support of this speculation, participants indicated ameliorated body (dis)satisfaction following letter writing regardless of condition. Other studies that reported a significant benefit of self-compassion compared it to a wait-list [14,15] or true (i.e., no intervention) [56] control rather than an active control. Perhaps the present study’s “neutral” control condition, (i.e., daily activity letter) actually served as a distraction from body dissatisfaction. Women with negative body image and higher BMIs in another study experienced ameliorated affect and body (dis)satisfaction immediately after a distraction task [16], indicating that distraction may be a helpful short-term strategy in the face of body dissatisfaction. The positive thinking letter-writing exercise may have decreased the perceived importance of appearance as the women considered other important aspects of their lives. In fact, the positive thinking condition may have targeted self-esteem, as Moffitt and colleagues used a similar writing prompt (asking participants to reflect on their positive “attributes and accomplishments” [61] (p. 70) with the intention of increasing self-esteem.

The exploratory analyses indicated that the self-compassion letter-writing exercise improved body image and self-compassion among the subgroup with higher BMIs and WBI. Instead of experiencing critical thoughts about their weight, these individuals were prompted to think kindly and compassionately about their appearance, resulting in more balanced or adaptive thoughts regarding their body. In contrast, for those with low WBI, the positive thinking exercise promoted the greatest self-compassion and lowest negative affect. Given the negative relationship between WBI and self-compassion [34], the self-compassion exercise may not have provided a new outlook on body image for these participants. It may be that thinking about the positive aspects of one’s life and body acted as a specific type of self-kindness, working to increase an already self-compassionate mindset.

The results of this study should be considered in light of several limitations. First, the study sample consisted of undergraduate females attending a Canadian university and the results may not generalize to samples with different demographic characteristics, for example, varying ages or education levels. Second, as the mean population BMI is around 25 [41], using the BMI threshold of 25 for those in the “higher BMI” group, although consistent with typical BMI categories, means that our results may only reflect the experiences of those with “above average” BMI. The study should be replicated specifically with individuals who have higher weight than most others and who therefore may experience even more weight-based stigma. Third, most dependent variables were measured at one time point which precludes examining changes over time. Fourth, the I-PANAS-SF and the SCS were adapted to reflect the state-level variables that this study attempted to capture because a validated state-level measure of self-compassion did not exist.

Although the ethnic diversity of participants in the present study is a notable strength, future studies should recruit larger stratified samples from diverse ethnicities to compare the effect of self-compassion and WBI on body image given that body image may differ for individuals from different ethnic backgrounds (e.g., [62]). Another notable strength is the study’s focus on the higher BMI group. This group has been largely absent in the literature examining self-compassion interventions for body image; this represents a significant gap considering this group experiences higher-than-average body image concerns [25].

Engaging in daily meditations over a longer period [14], addressing each component of self-compassion in a four-page writing exercise [20], and viewing self-compassionate quotes on social media platforms [24] are all strategies that have been shown to increase self-compassion in comparison to control groups. In future research, the self-compassion exercise used in the current study could be modified by increasing the letter length, asking participants to complete multiple self-compassion exercises over an extended period, or adding self-compassionate meditations to better induce self-compassion. A recent pilot study examined the feasibility and acceptability of a three-week-long self-compassion intervention for women with higher BMIs and WBI [26]. The current results would predict a positive response to this intervention given the benefits of the self-compassion exercise on positive body image for individuals with higher BMI and WBI. In line with the current results, this pilot study found that their self-compassion intervention promoted more body appreciation but did not seem to decrease body image shame [26].

To increase ecological validity, self-compassion strategies could be developed for women to practice in the face of body dissatisfaction that arises throughout their day-to-day lives. Clarifying the nature of the interaction between WBI and self-compassion in predicting body image for individuals with higher BMIs would also be a helpful next step. Further, investigating self-compassion as an intervention strategy for targeting WBI may aid in directly reducing these harmful self-stigmas. Finally, the investigation of WBI as a factor influencing the outcome of body image interventions is a novel contribution to the literature which could have important clinical implications. For example, knowledge that individuals with different levels of WBI may respond differently to certain types of body-image interventions could prompt the development of WBI screening tools to inform the selection of preferred body-image interventions for individuals with higher BMIs.

## 5. Conclusions

Self-compassion letter-writing may have benefits for body image among females with higher BMI and internalized weight bias. The relationship between self-compassion and internalized weight bias should be further examined to inform the development of effective interventions to improve body image among this subgroup given that most body image research has focused on females with smaller bodies.

## Figures and Tables

**Table 1:** Demographic Characteristics as a Function of Experimental Condition and Weight Group.

Variable	Self-Compassion		Positive Thinking		Neutral Control	
	HBMI *	LBMI *	HBMI	LBMI	HBMI	LBMI
Age	22.45 (7.00)	19.87(2.32)	20.17 (3.23)	19.96 (3.38)	22.04 (6.74)	20.72 (7.11)
BMI	29.29 (2.75)	21.49(2.69)	28.93 (3.24)	21.40 (2.63)	29.68 (5.23)	21.52 (2.11)
Ethnic Group						
White	15 (46.88%)	10 (38.46%)	14 (50.00%)	10 (38.46%)	8 (28.57%)	10 (37.04%)
South Asian	4 (12.50%)	4 (15.38%)	5 (17.86%)	5 (19.23%)	8 (28.57%)	4 (14.81%)
East Asian	0 (0%)	8 (30.77%)	1 (3.57%)	8 (30.77%)	1 (3.57%)	5 (18.52%)
Black	4 (12.50%)	0 (0%)	5 (17.86%)	1 (3.85%)	2 (7.14%)	2 (7.41%)
Arabic/Middle Eastern	2 (6.25%)	0 (0%)	0 (0%)	1 (3.85%)	4 (14.29%)	1 (3.70%)
Hispanic/Latina	1 (3.13%)	0 (0%)	2 (7.14%)	0 (0%)	2 (7.14%)	0 (0%)
Indigenous	0 (0%)	0 (0%)	0 (0%)	0 (0%)	1 (3.57%)	0 (0%)
Other	6 (18.75%)	4 (15.38%)	1 (3.57%)	1 (3.85%)	2 (7.14%)	5 (18.52%)
Education Level						
1st year	21 (65.63%)	16 (61.54%)	17 (60.71%)	13 (50.00%)	17 (60.71%)	16 (59.26%)
2nd year	1 (3.13%)	3 (11.54%)	0 (0%)	3 (11.54%)	6 (21.43%)	3 (11.11%)
3rd year	5 (15.63%)	5 (19.23%)	5 (17.86%)	5 (19.23%)	1 (3.57%)	5 (18.52%)
4th year or beyond	5 (15.63%)	2 (7.69%)	6 (21.43%)	5 (19.23%)	4 (14.29%)	3 (11.11%)

* HBMI = higher BMI group; LBMI = lower BMI group. Results are presented as mean (standard deviation) for continuous variables and N (%) for categorical variables.

**Table 2:** Mean Scores (Standard Deviations) on Dependent Variables as a Function of Experimental Condition and Weight Group.

Variable	Self-Compassion		Positive Thinking		Neutral Control	
	HBMI *	LBMI *	HBMI	LBMI	HBMI	LBMI
Self-Compassion	3.34 (0.59)	3.48 (0.55)	3.40 (0.71)	3.31 (0.55)	3.07 (0.58)	3.25 (0.54)
Self-Esteem	61.85 (14.60)	65.00 (13.60)	63.17 (14.57)	64.41 (16.62)	58.04 (18.53)	62.01 (14.06)
Body Image	4.70 (1.36)	5.74 (1.45)	4.80 (1.30)	5.32 (1.31)	4.23 (1.81)	5.14 (1.31)
Positive Affect	15.65 (3.82)	15.04 (3.80)	16.75 (4.21)	15.00 (3.48)	15.41 (4.07)	13.58 (5.05)
Negative Affect	9.45 (3.51)	8.52 (4.23)	8.93 (4.25)	8.65 (4.64)	10.65 (5.08)	8.11 (3.32)

* HBMI = higher BMI group; LBMI = lower BMI group.

## Data Availability

The data that support the findings of this study are available upon request from the corresponding author; the data are not publicly available due to privacy or ethical reasons.

## References

[1] Rodin J., Silberstein L., Striegel-Moore R. (1984). Women and weight: A normative discontent. Neb. Symp. Motiv..

[2] Garner D.M., Garfinkel P.E., Schwartz D., Thompson M. (1980). Cultural expectations of thinness in women. Psychol. Rep..

[3] Bessenoff G.R. (2006). Can the media affect us? Social comparison, self-discrepancy, and the thin ideal. Psychol. Women Q..

[4] Fallon E.A., Hausenblas H.A. (2005). Media images of the “ideal” female body: Can acute exercise moderate their psychological impact?. Body Image.

[5] Harper B., Tiggemann M. (2008). The effect of thin ideal media images on women’s self-objectification, mood, and body image. Sex Roles.

[6] Thompson J.K., Stice E. (2001). Thin-ideal internalization: Mounting evidence for a new risk factor for body-image disturbance and eating pathology. Curr. Dir. Psychol. Sci..

[7] Stice E., Shaw H.E. (2002). Role of body dissatisfaction in the onset and maintenance of eating pathology: A synthesis of research findings. J. Psychosom. Res..

[8] Betz D.E., Ramsey L.R. (2017). Should women be “All About That Bass?”: Diverse body-ideal messages and women’s body image. Body Image.

[9] Fredrickson B.L., Roberts T.A. (1997). Objectification theory: Toward understanding women’s lived experiences and mental health risks. Psychol. Women Q..

[10] Rosewall J.K., Gleaves D.H., Latner J.D. (2018). Psychopathology factors that affect the relationship between body size and body dissatisfaction and the relationship between body dissatisfaction and eating pathology. Front. Psychol..

[11] Choi E., Choi I. (2016). The associations between body dissatisfaction, body figure, self-esteem, and depressed mood in adolescents in the United States and Korea: A moderated mediation analysis. J. Adolesc..

[12] Bornioli A., Lewis-Smith H., Smith A., Slater A., Bray I. (2019). Adolescent body dissatisfaction and disordered eating: Predictions of later risky health behaviours. Soc. Sci. Med..

[13] Mars B., Heron J., Klonsky E.D., Moran P., O’Conner R.C., Tilling K., Wilkinson P., Gunnell D. (2018). What distinguishes adolescents with suicidal thoughts from those who have attempted suicide? A population-based birth cohort study. J. Child Psychol. Psychiatry.

[14] Albertson E.R., Neff K.D., Dill-Shackleford K.E. (2014). Self-compassion and body dissatisfaction in women: A randomized controlled trial of a brief meditation intervention. Mindfulness.

[15] Toole A.M., Craighead L.W. (2016). Brief self-compassion meditation training for body image distress in young adult women. Body Image.

[16] David L.A. (2018). The Impact of Cognitive Restructuring and Self-Compassion Strategies on Negative Body Image among Women with Higher Body Weight: An Experimental Investigation. Unpublished Doctoral Dissertation.

[17] Forbes Y.N., Moffitt R.L., Van Bokkel M., Donovan C.L. (2020). Unburdening the weight of stigma: Findings from a compassion-focused group program for women with overweight and obesity. J. Cogn. Psychother..

[18] Neff K.D. (2003). The development and validation of a scale to measure self-compassion. Self Identity.

[19] De Souza L.K., Hutz C.S. (2016). Self-compassion in relation to self-esteem, self-efficacy, and demographic aspects. Paidéia.

[20] Przezdziecki A., Sherman K.A. (2016). Modifying affective and cognitive responses regarding body image difficulties in breast cancer survivors using a self-compassion-based writing intervention. Mindfulness.

[21] Ferreira C., Pinto-Gouveia J., Duarte C. (2013). Self-compassion in the face of shame and body image dissatisfaction: Implications for eating disorders. Eat. Behav..

[22] Breines J., Toole A., Tu C., Chen S. (2014). Self-compassion, body image, and self-reported disordered eating. Self Identity.

[23] Homan K.J., Tylka T.L. (2015). Self-compassion moderates body comparison and appearance self-worth’s inverse relationships with body appreciation. Body Image.

[24] Slater A., Varsani N., Diedrichs P.C. (2017). #fitspo or #loveyourself? The impact of fitspiration and self-compassion Instagram images on women’s body image, self-compassion, and mood. Body Image.

[25] Sira B., Ballard S.M. (2009). An ecological approach to examining body satisfaction in Caucasian and African American female college students. Fam. Consum. Sci. Res. J..

[26] Haley E.N., Dolbier C.L., Carels R.A., Whited M.C. (2022). A brief pilot self-compassion intervention for women with overweight/obesity and internalized weight bias: Feasibility, acceptability, and future directions. J. Context. Behav. Sci..

[27] Falkner N.H., French S.A., Jeffery R.W., Neumark-Sztainer D., Sherwood N.E., Morton N. (1999). Mistreatment due to weight: Prevalence and sources of perceived mistreatment in women and men. Obes. Res..

[28] Brochu P.M., Esses V.M. (2011). What’s in a name? The effects of the labels “fat” versus “overweight” on weight bias. J. Appl. Soc. Psychol..

[29] Lillis J., Luoma J.B., Levin M.E., Hayes S.C. (2010). Measuring weight self-stigma: The weight self-stigma questionnaire. Obesity.

[30] Durso L.E., Latner J.D., Ciao A.C. (2016). Weight bias internalization in treatment-seeking overweight adults: Psychometric validation and associations with self-esteem, body image, and mood symptoms. Eat. Behav..

[31] Pearl R.L., Puhl R.M. (2016). The distinct effects of internalizing weight bias: An experimental study. Body Image.

[32] Fekete E.M., Herndier R.E., Sander A.C. (2021). Self-compassion, internalized weight stigma, psychological well-being, and eating behaviors in women. Mindfulness.

[33] Roberto C.A., Sysko R., Bush J., Pearl R., Puhl R.M., Schvey N.A., Dovidio J.F. (2012). Clinical correlates of the Weight Bias Internalization Scale in a sample of obese adolescents seeking bariatric surgery. Obesity.

[34] Pullmer R., Kerrigan S.G., Grilo C.M., Lydecker J.A. (2021). Factors linking perceived discrimination and weight bias internalization to body appreciation and eating pathology: A moderated mediation analysis of self-compassion and psychological distress. Stigma Health.

[35] Hilbert A., Braehler E., Schmidt R., Lӧwe B., Hӓuser W., Zenger M. (2015). Self-compassion as a resource in the self-stigma process of overweight and obese individuals. Obes. Facts.

[36] Faul F., Erdfelder E., Buchner A., Lang A.-G. (2009). Statistical power analyses using G*Power 3.1: Tests for correlation and regression analyses. Behav. Res. Methods.

[37] Bardone-Cone A.M., Cass K.M., Ford J.A. (2008). Examining body dissatisfaction in young men within a biopsychosocial framework. Body Image.

[38] Cafri G., Thompson J.K. (2004). Measuring male body image: A review of the current methodology. Psychol. Men Masc..

[39] National Heart, Lung, and Blood Institute. (n.d.). Calculate Your BMI—Standard BMI Calculator. https://www.nhlbi.nih.gov/health/educational/lose_wt/BMI/bmicalc.htm.

[40] Humphreys S. (2010). The unethical use of BMI in contemporary general practice. Br. J. Gen. Pract..

[41] Nutall F.Q. (2015). Body Mass Index: Obesity, BMI, and health: A critical review. Nutr. Today.

[42] Pearl R.L., Puhl R.M. (2014). Measuring internalized weight attitudes across body weight categories: Validation of the Modified Weight Bias Internalization Scale. Body Image.

[43] Durso L.E., Latner J.D. (2008). Understanding self-directed stigma: Development of the Weight Bias Internalization Scale. Obesity.

[44] Abend R., Dan O., Maoz K., Raz S., Bar-Haim Y. (2014). Reliability, validity, and sensitivity of a computerized visual analog scale measuring state anxiety. J. Behav. Ther. Exp. Psychiatry.

[45] Heinberg L.J., Thompson J.K. (1995). Body image and televised images of thinness and attractivess: A controlled laboratory investigation. J. Soc. Clin. Psychol..

[46] Garner D.M., Olmsted M.P., Polivy J. (1983). Development and validation of a multidimensional eating disorder inventory for anorexia nervosa and bulimia. Int. J. Eat. Disord..

[47] Thompson E.R. (2007). Development and validation of an internationally reliable short-form of the positive and negative affect schedule (PANAS). J. Cross-Cult. Psychol..

[48] Watson D., Clark L.A., Tellegen A. (1998). Development and validation of brief measures of positive and negative affect: The PANAS scales. J. Personal. Soc. Psychol..

[49] Cash T.F., Fleming E.C., Alindogan J., Steadman L., Whitehead A. (2002). Beyond body image as a trait: The development and validation of the Body Image States Scale. Eat. Disord..

[50] Heatherton T.F., Polivy J. (1991). Development and validation of a scale for measuring state self-esteem. J. Personal. Soc. Psychol..

[51] Helm L. (2016). The Effect of a Brief Self-Compassion Intervention on Emotion Regulation in Individuals with Generalized Anxiety Disorder. Ph.D. Dissertation.

[52] Gonen M.L. (2015). Does the Induction of Self-Compassion Buffer Negative Affect in Those Exposed to Social Evaluation?. Ph.D. Dissertation.

[53] Ziemer K.S., Lamphere B.R., Raque-Bogdan T.L., Schmidt C.K. (2019). A randomized controlled study of writing interventions on college women’s positive body image. Mindfulness.

[54] Kelly A.C., Waring S.V. (2018). A feasibility study of a 2-week self-compassionate letter-writing intervention for nontreatment seeking individuals with typical and atypical anorexia nervosa. Int. J. Eat. Disord..

[55] Field A. (2018). Discovering Statistics Using IBM SPSS Statistics.

[56] Leary M.R., Tate E.B., Adams C.E., Allen A.B., Hancock J. (2007). Self-compassion and reactions to unpleasant self-relevant events: The implications of treating oneself kindly. J. Personal. Soc. Psychol..

[57] Seekis V., Bradley G.L., Duffy A. (2017). The effectiveness of self-compassion and self-esteem writing tasks in reducing body image concerns. Body Image.

[58] Stern N.G., Engeln R. (2018). Self-compassionate writing exercises increase college women’s body satisfaction. Psychol. Women Q..

[59] Boyraz G., Legros D.N., Berger W.B. (2020). Self-criticism, self-compassion, and perceived health: Moderating effect of ethnicity. J. Gen. Psychol..

[60] Lockard A.J., Hayes J.A., Neff K., Locke B.D. (2014). Self-compassion among college counseling centre clients: An examination of clinical norms and group differences. J. Coll. Couns..

[61] Moffitt R.L., Neumann D.L., Williamson S.P. (2018). Comparing the efficacy of a brief self-esteem and self-compassion intervention for state body dissatisfaction and self-improvement motivation. Body Image.

[62] Grabe S., Hyde J.S. (2006). Ethnicity and body dissatisfaction among women in the United States: A meta-analysis. Psychol. Bull..

